# Association of the Transcription Factor 7 Like 2 (TCF7L2) Polymorphism With Diabetic Nephropathy Risk: A Meta-Analysis

**DOI:** 10.1097/MD.0000000000003087

**Published:** 2016-03-18

**Authors:** Zhenqian Fan, Qiliang Cai, Yu Chen, Xuying Meng, Fenglin Cao, Shaoxiong Zheng, Jianchao Guo

**Affiliations:** From the Department of Endocrinology, Second Hospital of Tianjin Medical University (ZF, YC, XM, FC, SZ, JG); and Department of Urology, Second Hospital of Tianjin Medical university (QC), Tianjin, Republic of China.

## Abstract

It is assumed that genetic factors may participate in the development of diabetic nephropathy (DN). The association between *TCF7L2* gene polymorphism and DN risk is still unclear. To evaluate the relationship, we performed this meta-analysis.

Eligible relevant studies were searched and selected from PubMed, Embase, and ISI Web of Science. Summary effect estimates were derived using a random effects model, with attention to study quality and publication bias. Ethnical approval was not necessary, because this meta-analysis was based on published articles, and did not involve patient consent.

A total of 7 studies were identified. Analysis of all studies indicated significant association between *TCF7L2* gene polymorphism and DN risk (odds ratio [OR] = 1.31, 95% confidence interval (CI) = 1.10–1.56, *P*_heterogeneity_ < 0.00001, *P* = 0.002). Subgroup analysis showed similar results in Asian (OR = 1.33, 95% CI = 1.10–1.62, *P*_heterogeneity_ = 0.03, *P* = 0.004), in Caucasian (OR = 2.27, 95% CI = 1.78–2.90, *P*_heterogeneity_ = 0.17, *P* < 0.00001), in rs7903146 mutation (OR = 1.61, 95% CI = 1.25–2.07, *P*_heterogeneity_ < 0.00001, *P* = 0.0002), However, no association was observed in Negroid (OR = 1.10, 95% CI = 0.90–1.35, *P*_heterogeneity_ < 00001, *P* = 0.36).

Our results suggest that *TCF7L2* gene polymorphism may contribute to the risk of DN. However, more studies should be launched in the future.

## INTRODUCTION

The incidence and prevalence of diabetes mellitus (DM) have increased sharply throughout the world. According to the latest report, the prevalence of DM has increased to 11.7% in China^1^ and 9.1% in the world.^2^ The total number of adult diabetes is expected to rise to 642 million by 2040.^2^ Diabetic nephropathy (DN), 1 of the most serious complications of DM, is a progressive kidney disease caused by damage to the capillaries in the kidneys’ glomeruli. End-stage renal disease (ESRD) is the most severe form of chronic kidney disease, also known as Stage 5 chronic kidney disease or kidney failure. DN is the leading cause of ESRD in the Europe, Japan, and the United States.^3^ DN was also the major cause for 40.4% of newly developed ESRD in the United States.^4,5^ In the UK, it was estimated that around one third of patients with ESRD were due to diabetes.^6^ In Korea, according to the Fifth Korea National Health and Nutrition Examination Survey in 2011, the prevalence of DN was 26.7%.^7^ In China, the Shanghai Diabetic Complications Study reported that the prevalence of DN was 26.2%.^8^ The expenditure for people with DN are extraordinarily high. In the Medicare population alone, DN-related expenditures among this mostly older group were nearly $25 billion in 2011.^9^ Therefore, earlier diagnosis and prevention of DN become imminent. However, the mechanism of DN is complicated, in addition to the risk of poor glycemic control and hypertension, inherited factors are considered to play an important role in its progression.

Transcription factor 7-like 2 (*TCF7L2*) gene is located on chromosome 10q25.3,^10^ which encodes a transcription factor with a high-mobility box and functions in gene activations related to downstream events of the Wnt signaling pathway. TCF7L2 has been revealed as an susceptibility gene for type 2 diabetes.^11^ Selective disruption of TCF7L2 in the pancreatic β cell impairs secretory function and lowers β cell mass.^12^ Clinical studies suggested that *TCF7L2* gene impaired glucose tolerance through effects on glucagon as well as insulin secretion.^13^ Several meta-analysis^14–18^ also concluded that *TCF7L2* gene polymorphisms were associated with DM.

However, the relationship between TCF7L2 polymorphism and DN is inconclusive. Based on their studies, Bodhini et al^19^ pointed out that association between *TCF7L2* gene polymorphism and DN was mediated through diabetes. Results of a family-based study indicated no association between *TCF7L2* gene and the increased risk for development of chronic kidney disease in non-diabetic subjects.^20^ But Buraczynska et al^21^ argued that *TCF7L2* gene polymorphism conferred the risk of developing DN. Similarly, studies on renal transplant patients also suggested TCF7L2 was strongly and independently associated with posttransplant diabetes mellitus.^22^ Furthermore, Araoka et al^23^ did a systemic work, they found that advanced glycation end products (AGEs) induced TCF7L2 expression through transforming growth factor-β (TGF-β), and TGF-β could induce TCF7L2 translocation from the cytoplasm to the nucleus. Then, TCF7L2 bounded to the activin receptor-linked kinase1 (ALK1) promoter, resulting in increased ALK1 expression. Increased ALK1 enhanced the effect of TGF-β and further promoted the phosphorylation of Smad1. These interactions caused the phenotypic changes in mesangial cells, thereby resulting in the development of glomerulosclerosis. They concluded that the AGEs/TGF-β/TCF7L2/ALK1/Smad1 signaling pathway plays a key role in the development of DN. In order to draw accurate conclusion, we performed this meta-analysis.

## MATERIALS AND METHODS

### Selection of Eligible Studies

We searched and selected from PubMed, Embase, and ISI Web of Science. Last search was updated on October 2015, the following terms were used: DM, DN, *TCF7L2*, gene polymorphism, including all alternative locations and combinations of the terms. In addition, hand searching was also performed on the reference lists of all included studies as well as reviews recently published. If there were several papers coming from the same study, only the most recent or complete study was included. Each potentially eligible study was evaluated independently by 2 authors (Fan and Meng).

### Inclusion and Exclusion Criteria

Two authors (Fan and Meng) searched and selected the eligible studies, during the process, the following inclusion criteria were used: firstly, the studies should be designed as case-control or cohort study. Secondly, in the studies, there should be sufficient genotype data to calculate 95%confidence intervals (CI) for risk ratios or Odds ratios (ORs). Thirdly, last but the most important is there should be an appropriate description of TCF7L2 polymorphisms in DN patients and controls in these eligible articles. We excluded some studies, the exclusion criteria include studies on animals; studies without the raw data of genotype of TCF7L2; and case reports, editorials, and review articles.

### Data Extraction and Quality Assessment

For each included studies, these data were extracted, including the first author, year of publication, country or area, ethnicity, characteristics, allele frequencies with TCF7L2 genotype in DN patients, and controls. We did not exclude certain studies because of the limited number of cases. This meta-analysis was guided to conduct by the PRISMA statement for preferred reporting of systematic reviews and meta-analysis.^24^

### Data Synthesis and Analysis

We used ORs with 95% CIs for genotypes and alleles to assess the strength of association between *TCF7L2* gene polymorphism and DN risk. Allele frequencies were estimated, the ORs were conducted for the allele contrast. Heterogeneity was evaluated using *I*^2^ statistic, which takes values from 0% to 100% with higher values indicating greater degree of heterogeneity. The value greater than 50% was considered as significant heterogeneity. If there was significant heterogeneity, the random effects model would be used to analyze the pooled ORs^25^ or analyzed by the fixed effects model.^26^ To discern the potential reasons of heterogeneity, subgroup analyses were performed by group studies with similar characteristic, such as ethnicity and the same loci rs7903146.

The sensitivity analyses were performed to evaluate the reliability of the results by excluding 1 study each time and rerunning the analysis. An estimate of potential publication bias was carried out by inverted funnel plot, Egger test, and Begg test. All statistical tests were performed by RevMan software (version5.3, Review Manager, Copenhagen: The Nordic Cochrane Centre, The Cochrane Collaboration, 2014). Sensitivity analysis, Begg test, and Egger test were accomplished by Stata (version11.2, StataCorp, College Station, TX). All the *P* values were 2-sided.

## RESULTS

### Characteristics of Included Studies

**FIGURE 1:**
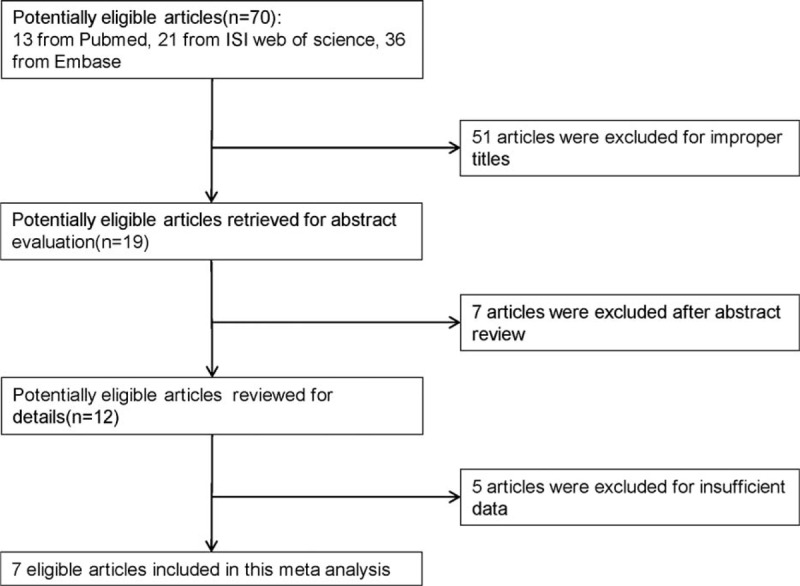
Flow diagram for study selection process.

The process of study selection for inclusion in this meta-analysis is present in Figure 1. Seventy relevant articles were identified from PubMed, Embase, and ISI Web of Science. Among these articles, 51 were excluded for improper titles, 7 were excluded after review the abstract, 5^27–31^ were excluded because of insufficient data. Overall, 7^19,21,32–36^ articles were included in our meta-analysis. All the included articles were case-control studies. We calculated the allele frequencies in the DN group and control group. The minor allele frequencies were 3815 in DN group, 4303 in control group, total allele frequencies were 11,698 in DN group, and 16,034 in control group. Of the 7 studies, there were three^19,32,35^ with Asian ethnicity, two^21,33^ with Caucasian ethnicity, two^34,36^ with Negroid, respectively. Six articles^21,32–36^ probed the association of rs7903146 mutation and DN risk. Main characteristics for all eligible studies were shown in Table 1.

**TABLE 1:**
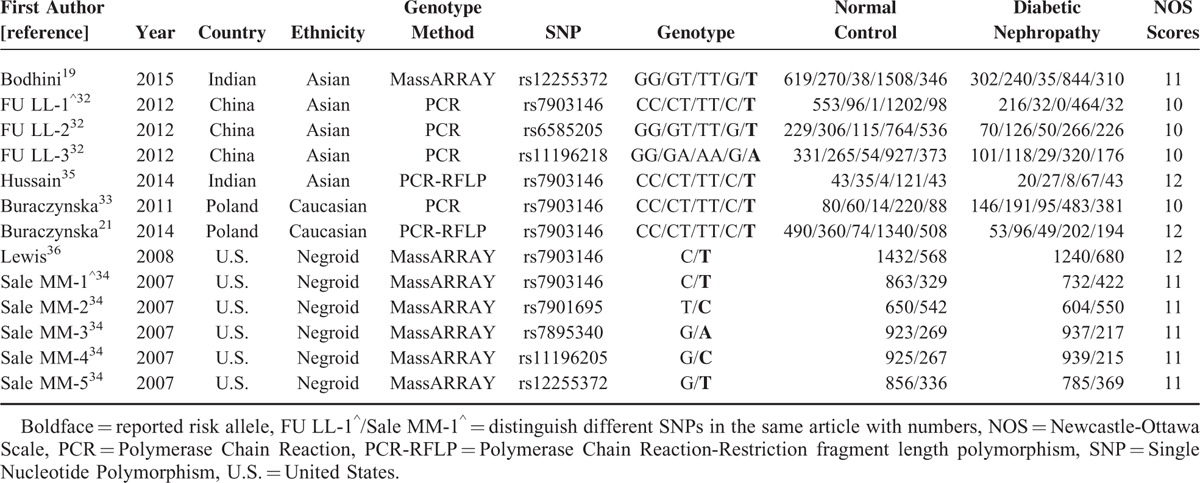
Association of TCF7L2 Polymorphism with DN risk

### Meta-Analysis Results

**FIGURE 2:**
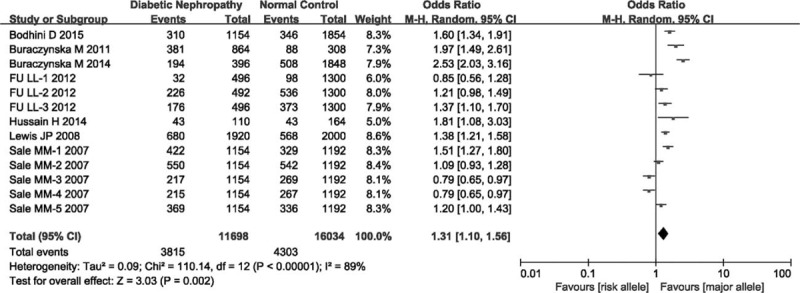
Forest plot for the overall association between TCF7L2 and DN risk.

**FIGURE 3:**
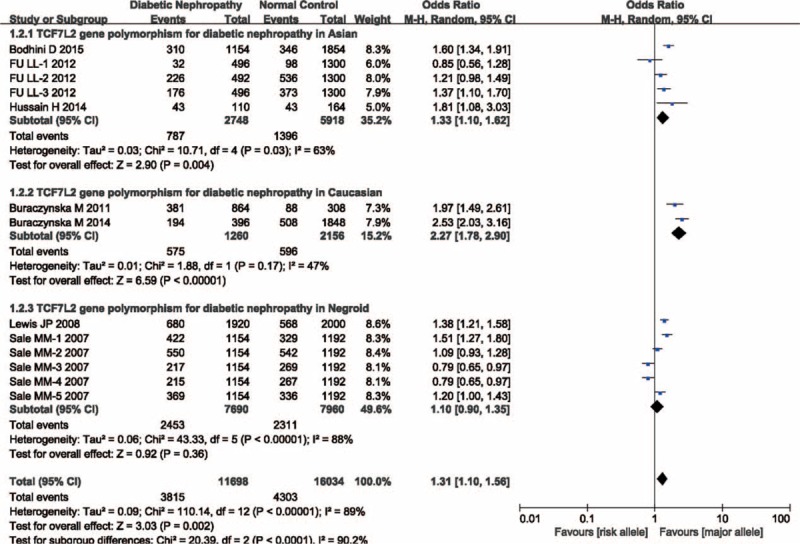
Subgroup analysis performed by ethnicity.

**FIGURE 4:**
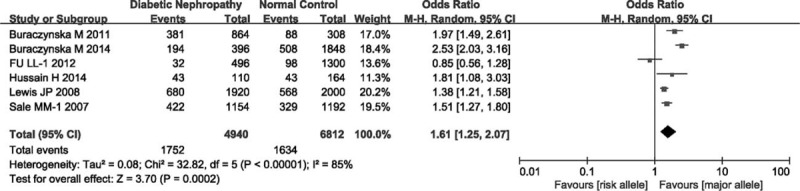
Forest plot for the association between TCF7L2 rs7903146 mutation and DN risk.

Aggregated ORs and heterogeneity test results for the association of TCF7L2 polymorphism and DN risk were shown in Figure 2. In total, there was significant association between TCF7L2 polymorphism and DN risk (OR = 1.31, 95% CI = 1.10–1.56, *P*_heterogeneity_ < 0.00001, *P* = 0.002). The estimate of *I*^2^ showed significant heterogeneity (*I*^2^ = 89%) among the studies, therefore, we used random effects model in this meta-analysis. To discern the potential reasons of heterogeneity, subgroup analyses were performed. Stratified analysis by ethnicities, significant association was observed in Asian (OR = 1.33, 95% CI = 1.10–1.62, *P*_heterogeneity_ = 0.03, *P* = 0.004), and in Caucasian (OR = 2.27, 95% CI = 1.78–2.90, *P*_heterogeneity_ = 0.17, *P* < 0.00001), but not in Negroid (OR = 1.10, 95% CI = 0.90–1.35, *P*_heterogeneity_ < 0.00001, *P* = 0.36; Figure 3). Subgroup analysis of rs7903146 mutation suggested there was significant association (OR = 1.61, 95% CI = 1.25–2.07, *P*_heterogeneity_ < 0.00001, *P* = 0.0002; Figure 4).

### Test of Heterogeneity

There was significant heterogeneity among the included studies in this meta-analysis. After evaluating the reasons of heterogeneity by ethnicities, rs7903146, however, there was still significant heterogeneity among the studies.

### Sensitivity Analysis

Finally, we performed sensitivity analysis by excluding 1 study each time and recalculated the pooled OR for the remainder of the studies. Some pooled ORs were effectively altered in the sensitivity analysis, but most of the pooled ORs were not materially changed, suggesting that our results were credible.

### Publication Bias

**FIGURE 5:**
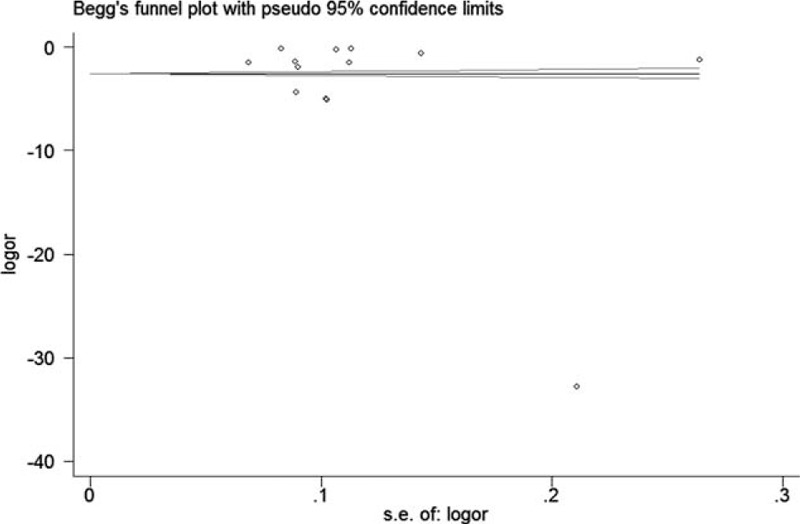
Begg test was held for the detection of publication bias.

**FIGURE 6:**
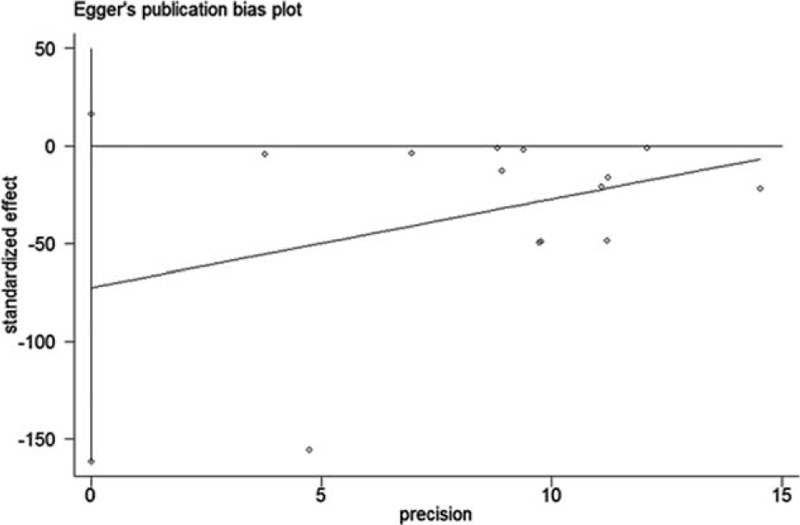
Egger test was held for the detection of publication bias.

Begg funnel plot and Egger test were performed to assess the publication bias. No evidence of publication bias was detected in Begg test (*P* = 0.246) (Figure 5) and Egger test (*P* = 0.100) (Figure 6).

## DISCUSSION

Recent epidemiological studies indicate that the prevalence of DM has increased to 11.7% in China and 9.1% in the world, respectively. According to the latest report, the total number of adult diabetes have increased to 415 million by 2015.^2^ DN is 1 of the most common microvascular complications of DM, and is the leading cause of ESRD in some countries. The pathogenesis of DN is complicated, besides poor glycemic control and hypertension, genetic susceptibility also play an important role. TCF7L2, also known as transcription factor 4 , has been revealed as a susceptibility gene for type 2 diabetes.^11^ However, the association between *TCF7L2* gene and DN risk is elusive. To derive a more precise estimation of the relationship, we performed this meta-analysis.

Our result provided a new evidence that *TCF7L2* gene polymorphism was associated with DN risk. In the subgroup analysis by ethnicities, the result indicated that *TCF7L2* gene polymorphism was associated with DN risk in Asian and Caucasian, but not in Negroid. We presumed that the limited studies and sample size of subject might be the reason on this inconsistency. Rs7903146 mutation was associated with DM,^37,38^ and our subgroup analysis suggested that rs7903146 was also significant associated with DN. This was consistent with conclusions from FU et al^32^ and Buraczynska et al.^21^ Accordingly, we presumed that the rs7903146 mutation was important in the DM and DN, and the intervention of this mutation might bring new therapeutic strategy for DM and DN.

However, some limitations should be acknowledged in our meta-analysis. Firstly, DN is a continuous process which includes several different stages, from microalbuminuria to ESRD. Because of insufficient data, we did not probe subgroup analysis by different stages of DN, thus, we did not know whether TCF7L2 plays a different role in different stages, or how much influence our results. Secondly, the mechanism of DN is complicated; many factors may participate in its development, such as hypertension, poor glycemic control, diabetes duration, smoking, age, and others. Though we probed subgroup analysis, we still could not exclude all the potential influence factors. Thirdly, strong heterogeneities among the included studies made it less robust to interpret results. Finally, publication bias was found when assessing linear association between TCF7L2 polymorphism and DN risk.

Notwithstanding the limitations discussed above, our analysis still includes most of the published literature referred to TCF7L2 and DN. The results provide new evidence on that *TCF7L2* gene polymorphism may contribute to the risk of DN, although whether the association is direct or indirect still cannot be determined. Anyhow, this finding provides a new point of view on the mechanism of DN, and could be a direction for future research.
